# Impact of Adjuvant Chemotherapy on Variant Histology of Upper Tract Urothelial Carcinoma: A Propensity Score-Matched Cohort Analysis

**DOI:** 10.3389/fonc.2022.843715

**Published:** 2022-04-22

**Authors:** Chi-Wen Lo, Wei-Ming Li, Hung-Lung Ke, Yi-Huei Chang, Hsi-Chin Wu, I-Hsuan Alan Chen, Jen-Tai Lin, Chao-Yuan Huang, Chung-Hsin Chen, Jen-Shu Tseng, Wun-Rong Lin, Yuan-Hong Jiang, Yu-Khun Lee, Chung-You Tsai, Shiu-Dong Chung, Thomas Y. Hsueh, Allen W. Chiu, Yeong-Chin Jou, Ian-Seng Cheong, Yung-Tai Chen, Jih-Sheng Chen, Bing-Juin Chiang, Chih-Chin Yu, Wei Yu Lin, Chia-Chang Wu, Chuan-Shu Chen, Han-Yu Weng, Yao-Chou Tsai

**Affiliations:** ^1^ Division of Urology, Department of Surgery, Taipei Tzuchi Hospital, The Buddhist Tzu Chi Medical Foundation, New Taipei City, Taiwan; ^2^ School of Medicine, Buddhist Tzu Chi University, Hualien, Taiwan; ^3^ Department of Urology, Kaohsiung Medical University Hospital, Kaohsiung, Taiwan; ^4^ Department of Urology, School of Medicine, College of Medicine, Kaohsiung Medical University, Kaohsiung, Taiwan; ^5^ Graduate Institute of Medicine, College of Medicine, Kaohsiung Medical University, Kaohsiung, Taiwan; ^6^ Department of Urology, Ministry of Health and Welfare Pingtung Hospital, Pingtung, Taiwan; ^7^ Cohort Research Center, Kaohsiung Medical University, Kaohsiung, Taiwan; ^8^ Department of Urology, China Medical University Hospital, Taichung, Taiwan; ^9^ School of Medicine, China Medical University, Taichung, Taiwan; ^10^ Department of Urology, China Medical University Beigang Hospital, Yunlin, Taiwan; ^11^ Division of Urology, Department of Surgery, Kaohsiung Veterans General Hospital, Kaohsiung, Taiwan; ^12^ Department of Urology, National Taiwan University Hospital, College of Medicine, and National Taiwan University, Taipei, Taiwan; ^13^ Department of Urology, MacKay Memorial Hospital, Taipei, Taiwan; ^14^ Department of Medicine, Mackay Medical College, Taipei, Taiwan; ^15^ Institute of Biomedical Informatics, National Yang Ming Chiao Tung University, Taipei, Taiwan; ^16^ Department of Urology, Hualien Tzu Chi Hospital, Buddhist Tzu Chi Medical Foundation and Tzu Chi University, Hualien, Taiwan; ^17^ Division of Urology, Department of Surgery, Far Eastern Memorial Hospital, New Taipei City, Taiwan; ^18^ Department of Healthcare Information and Management, Ming Chuan University, Taoyuan, Taiwan; ^19^ Department of Nursing, College of Healthcare and Management, Asia Eastern University of Science and Technology, New Taipei City, Taiwan; ^20^ General Education Center, Eastern University of Science and Technology, New Taipei City, Taiwan; ^21^ Division of Urology, Department of Surgery, Taipei City Hospital Renai Branch, Taipei, Taiwan; ^22^ Department of Urology, School of Medicine, National Yang Ming Chiao Tung University, Taipei, Taiwan; ^23^ College of Medicine, National Yang Ming Chiao Tung University, Taipei, Taiwan; ^24^ Department of Urology, Ditmanson Medical Foundation Chiayi Christian Hospital, Chiayi, Taiwan; ^25^ Department of Health and Nutrition Biotechnology, Asian University, Taichung, Taiwan; ^26^ Department of Urology, Taiwan Adventist Hospital, Taipei, Taiwan; ^27^ College of Medicine, Fu-Jen Catholic University, New Taipei City, Taiwan; ^28^ Department of Urology, Cardinal Tien Hospital, New Taipei City, Taiwan; ^29^ Department of Life Science, College of Science, National Taiwan Normal University, Taipei, Taiwan; ^30^ Division of Urology, Department of Surgery, Chang Gung Memorial Hospital, Chia-Yi, Taiwan; ^31^ Chang Gung University of Science and Technology, Chia-Yi, Taiwan; ^32^ Department of Medicine, Chang Gung University, Taoyuan, Taiwan; ^33^ Department of Urology, Shuang Ho Hospital, Taipei Medical University, New Taipei City, Taiwan; ^34^ Taipei Medical University Research Center of Urology and Kidney (TMU-RCUK), Taipei Medical University, Taipei, Taiwan; ^35^ Division of Urology, Department of Surgery, Taichung Veterans General Hospital, Taichung, Taiwan; ^36^ Institute of Medicine, Chung Shan Medical University, Taichung City, Taiwan; ^37^ Department of Senior Citizen Service Management, National Taichung University of Science and Technology, Taichung City, Taiwan; ^38^ Department of Urology, National Cheng Kung University Hospital, College of Medicine, National Cheng Kung University, Tainan, Taiwan

**Keywords:** adjuvant chemotherapy, nephroureterectomy, upper urinary tract urothelial cancer, variant histology, UTUC

## Abstract

**Background:**

The advantage of adjuvant chemotherapy for upper urinary tract urothelial cancer (UTUC) has been reported, whereas its impact on upper tract cancer with variant histology remains unclear. We aimed to answer the abovementioned question with our real-world data.

**Design, Setting, and Participants:**

Patients who underwent radical nephroureterectomy (RNU) and were confirmed to have variant UTUC were retrospectively evaluated for eligibility of analysis. In the Taiwan UTUC Collaboration database, we identified 245 patients with variant UTUC among 3,109 patients with UTUC who underwent RNU after excluding patients with missing clinicopathological information.

**Intervention:**

Those patients with variant UTUC were grouped based on their history of receiving adjuvant chemotherapy or not.

**Outcome Measurements and Statistical Analysis:**

Propensity score matching was used to reduce the treatment assignment bias. Multivariable Cox regression model was used for the analysis of overall, cancer-specific, and disease-free survival.

**Results and Limitations:**

For the patients with variant UTUC who underwent adjuvant chemotherapy compared with those without chemotherapy, survival benefit was identified in overall survival in univariate analysis (hazard ratio (HR), 0.527; 95% confidence interval (CI), 0.285–0.973; *p* = 0.041). In addition, in multivariate analysis, patients with adjuvant chemotherapy demonstrated significant survival benefits in cancer-specific survival (OS; HR, 0.454; CI, 0.208–0.988; *p* = 0.047), and disease-free survival (DFS; HR, 0.324; 95% CI, 0.155–0.677; (*p* = 0.003). The main limitations of the current study were its retrospective design and limited case number.

**Conclusions:**

Adjuvant chemotherapy following RNU significantly improved cancer-related survivals in patients with UTUC with variant histology.

## Introduction

Upper urinary tract urothelial carcinoma (UTUC) is a rare cancer and accounts for only 5%–10% of genitourinary urothelial cancers (UC) in Western countries (1). UTUC with nontransitional-cell variant histopathology (vUTUC) is an even rarer situation, accounting for 8%~24% among historical UTUC series where the patients were managed with radical nephroureterectomy (RNU) (1–9). vUTUC is commonly associated with more adverse pathological features and advanced disease status at presentation when compared with those of UTUC with pure urothelial pathology (pUTUC) (1–4, 6–9). In addition, patients with vUTUC are usually associated with worse outcomes in overall (OS), cancer-specific (CSS), and disease-free survival (DFS) (1). Therefore, a variant histopathologic feature in UTUC is an important prognostic factor that should be recognized for subsequent treatment planning and disease surveillance.

Bladder UC shares several similar histopathological and prognostic features with UTUC. Variant histology in bladder UC is also associated with adverse pathological features and poor outcomes (10–12). The poor outcomes of variant histology bladder UC raised the speculation of whether perioperative chemotherapy will improve outcomes in this distinct subset of bladder UC. Neoadjuvant chemotherapy has been reported to improve survival outcomes with variant histologic features in bladder UC (10, 13). A recent meta-analysis revealed that good outcomes are associated with chemotherapy for small-cell and micropapillary variants, while chemotherapy has a potential role in squamous cell and adenocarcinoma variants (13).

The success of adjuvant chemotherapy in variant bladder UC certainly led to the speculation whether vUTUC would benefit from adjuvant chemotherapy, but the study of the more aggressive vUTUC is usually limited by its even rare presentation. Although the safety and efficacy of adjuvant chemotherapy for UTUC had been confirmed in the Perioperative Chemotherapy Versus Surveillance in Upper Tract urothelial cancer) trial, the outcomes of adjuvant chemotherapy for patients with vUTUC remain scarce (14). There is no randomized controlled trials (RCTs) or comparative trial focusing on the even rarer and more aggressive vUTUC. Therefore, we conducted a propensity score-matched study to examine the impact of adjuvant chemotherapy on vUTUC.

## Material and Methods

### Data Source

This UTUC registry database was conducted by the Taiwan UTUC Collaboration Group, a multicenter Internet-based registry partially sponsored by the Taiwan Urological Association. This retrospective study was reviewed and approved by the institutional review board (IRB No.: 063-X34-105), and the requirement of informed consent was waived due to its anonymous nature without any identifiable information in the database. The study protocols and methods were carried out in accordance with relevant guidelines and regulations.

### Patient Selection

**Figure 1:**
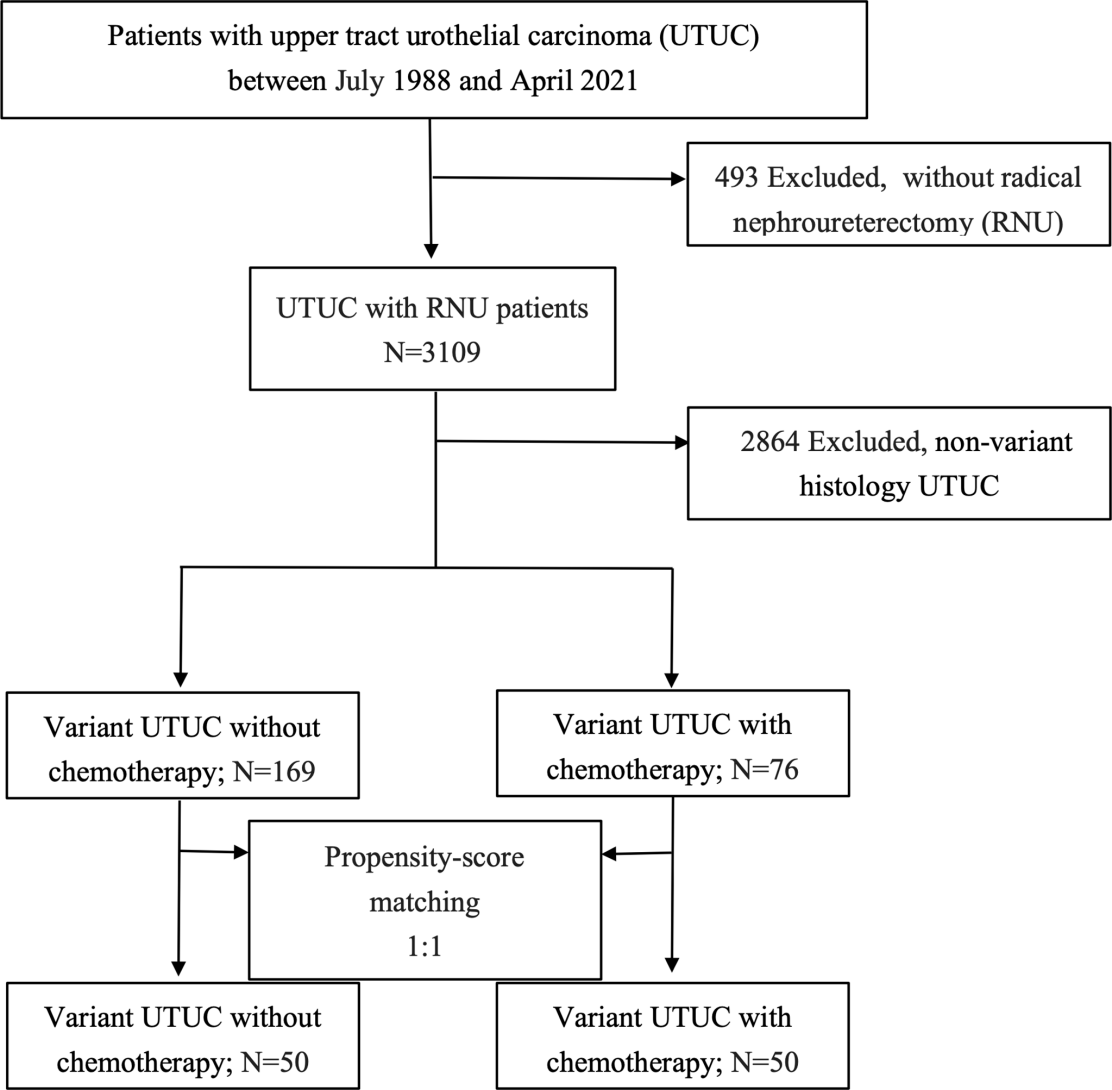
Flow diagram of case recruitment process.

Patients who underwent RNU and were confirmed to have UTUC with variant histology were retrospectively evaluated for eligibility of analysis. Those patients with postoperative adjuvant chemotherapy were defined as systemic chemotherapy given within 4 months after RNU (15). There were 3,109 patients examined for eligibility, with 245 cases enrolled for final analysis (**Figure 1**). vUTUC was defined as those patients with upper urinary tract carcinoma who exhibited a nontransitional-cell histopathology type under pathological evaluation. The groups were categorized by vUTUC with adjuvant chemotherapy (vUTUC+C/T) and vUTUC without adjuvant chemotherapy (vUTUC–C/T). Those cases with neoadjuvant chemotherapy or missing histology/staging information, chemotherapeutic agents, and survival information were excluded.

### Pathological Evaluation

The histopathological diagnosis and staging of RNU specimens were reviewed by local genitourinary pathologists in each institution according to the 2010 American Joint Committee on Cancer (AJCC) tumor-node-metastasis (TNM) staging system, and histopathologic grading was made according to the 2015 WHO/ISUP recommendation grading system. The histopathological diagnosis of variant histology has been well accepted by the uropathological community, and the diagnostic criteria were described in the WHO classification of tumors (16).

### Follow-Up

The follow-up schedule for patients was every 3–6 months in the 1st year after RNU and every 6–12 months thereafter. Cross-sectional imaging (computed tomography (CT) or/and magnetic resonance images (MRI)) was used to determine recurrence/progression-free statuses. Cystoscopy examination was used to determine urinary bladder recurrence. UTUC recurrence or metastasis was defined as local recurrence of tumor bed, regional lymph nodes, or distant metastasis.

### Matching Methods

To address the imbalance of potential confounders between the control and treatment adjuvant groups, we matched treatment “adjuvant” groups using propensity scores. In the statistical analysis of observational data, propensity score matching (PSM) is a statistical matching technique that attempts to estimate the effect of a treatment, policy, or other intervention by accounting for the covariates that predict receiving the treatment. In each simulated dataset, we estimated the propensity score using a logistic regression model to regress treatment status on the baseline covariates. The propensity score model included lymphovascular invasion, surgical margin, and pathological stage. We then formed matched pairs between the control group managed by watch and wait and those who had treatment adjuvant using nearest-neighbor matching gender (tolerance levels: 0), age (tolerance levels: 5), and propensity score (tolerance levels: 0.01). Our study on propensity score matching is one-to-one or pair matching, in which pairs of control and treated subjects are formed, such that matched subjects have similar values of the propensity score.

### Statistical Analysis

Demographic and clinicopathological differences between groups were compared using Pearson Chi-square with Bonferroni correction for categorical variables. The Kaplan–Meier estimator was used to estimate the rates of survival outcomes, and the survival curves were compared using the stratified log-rank test. Cox proportional hazard model was selected to assess the effect of the treatment groups on the survival outcomes, alone and after adjusting for potential confounders. Those clinicopathological variables were selected with stepwise regression then included in the multivariate analysis. All the univariate significant and nonsignificant relevant covariates should be put on the variable list to be selected. The significance levels for entry (SLE) and stay (SLS) are suggested to be set to 0.05 and 0.1, respectively. The best regression model is then identified manually by reducing the significant levels to 0:05, corresponding to the chosen level. All statistical assessments were two-tailed and considered statistically significant at *p* < 0.05. Statistical analyses were carried out with IBM SPSS statistical software version 22. The description of statistical methods was based on the standard format of statistical analysis of the Taiwan UTUC collaboration group.

## Results

### Study Cohort and Baseline Characteristics

In total, 245 patients who underwent RNU with variant histology were enrolled for final analysis. The recorded variant histopathological types included sarcomatoid differentiation (16.3%), squamous cell carcinoma (53.1%), adenocarcinoma (7.7%), neuroendocrine differentiation (4.1%), and mixed variant histology (7.7%). In total, 75 of 245 (30.6%) patients with vUTUC underwent adjuvant chemotherapy. **Table 1** summarizes the baseline clinical characteristics of the 169 cases in the vUTUC-C/T group and 75 cases in the vUTUC+C/T group before matching. Significant bias was observed in baseline characteristics in gender, age, comorbidity, tumor multiplicity, lymphovascular invasion status, pT/N stage, and preoperative renal function status before matching. After PSM, we derived 1:1 50 paired cohorts for vUTUC with and without adjuvant chemotherapy. These two groups were well-matched for all confounding variables (**Table 2**). In addition, there was no significant difference in the distribution of variant histology subtypes. The median cycles of adjuvant chemotherapy for the vUTUC+C/T group were 4 (interquartile range: 3–5). The most used chemotherapeutic regimen was gemcitabine plus cisplatin, followed by gemcitabine plus carboplatin, and then MVAC regimen (methotrexate, vinblastine, doxorubicin plus cisplatin). At the end of our follow-up, forty-three of 100 cases had an event in overall survival and 38 of 100 cases had at least one event in disease progression.

**Table 1:** Baseline demographic characteristics of patients with variant upper tract urothelial cancer (UTUC) undergoing radical nephroureterectomy (RNU) before matching.

Variables	UC with variants/no C/T (*N* = 169)	UC with variants/C/T (*N* = 75)	*p*-value^a^
	*N* (%)	*N* (%)	
Gender			
Men	56 (33.1)	45 (60.0)	<0.001^**^
Women	113 (66.9)	30 (40.0)	
Age			
<70	65 (38.5)	51 (68.0)	<0.001^**^
≥70	104 (61.5)	24 (32.0)	
Comorbidity			
Coronary artery disease	14 (8.3)	6 (7.0)	0.918
Arrhythmia	9 (5.3)	2 (2.0)	0.346
Hypertension	97 (57.4)	28 (36.0)	0.003^**^
Diabetes	51 (30.2)	18 (23.0)	0.296
Gouty arthritis	3 (1.8)	3 (3.0)	0.309
Gastrointestinal disorder	21 (12.4)	8 (10.0)	0.670
2nd malignancy (not urothelial cancer)	27 (16.0)	11 (14.0)	0.764
Tumor location			
Renal pelvis	87 (51.5)	35 (46.0)	0.593
Ureter	47 (27.8)	21 (27.0)	
Renal pelvis + ureter	35 (20.7)	20 (26.0)	
Tumor size			
<3 cm	56 (33.1)	26 (34.0)	0.869
≥3 cm	113 (66.9)	50 (65.0)	
RNU histology			
Low grade	5 (3.0)	0 (0.0)	0.499
High grade	158 (93.5)	73 (97.0)	
Gx	4 (2.4)	1 (1.0)	
G2	1 (0.6)	1 (1.0)	
Well-differentiated	1 (0.6)	0 (0.0)	
Multiplicity			
Not available	0 (0.0)	1 (1.0)	0.018^*^
No	115 (68.9)	39 (52.0)	
Yes	52 (31.1)	35 (46.0)	
CIS			
No	133 (78.7)	58 (76.0)	0.677
Yes	36 (21.3)	18 (23.0)	
Lymphovascular invasion			
No	112 (66.3)	38 (50.0)	0.016^*^
Yes	57 (33.7)	38 (50.0)	
Surgical margin			
Free	150 (88.8)	67 (88.0)	0.891
Positive	19 (11.2)	9 (11.0)	
Tumor necrosis			
No	108 (64.3)	44 (58.0)	0.403
Yes	60 (35.7)	31 (41.0)	
Synchronous bladder tumor			
No	142 (84.5)	61 (80.0)	0.684
Yes	26 (15.4)	15 (19.0)	
Pathological stage T			
pTis/pTa/pT0/pT1/pT2	58 (34.3)	10 (13.0)	0.001^**^
pT3/pT4	111 (65.7)	66 (86.0)	
Pathological stage N			
pN0	36 (21.3)	24 (31.0)	0.007^**^
pN+	20 (11.8)	17 (22.0)	
pNx	113 (66.9)	35 (46.0)	
eGFR			
≧60	44 (27.2)	33 (45.8)	0.005^**^
<60	118 (72.8)	39 (54.2)	
Post-OP eGFR			
≧60	13 (10.3)	13 (25.0)	0.012^*^
<60	113 (89.7)	39 (75.0)	
Histologic			
Sarcomatoid differentiation	27 (16.0)	13 (17.0)	0.687
Squamous cell carcinoma	86 (50.9)	44 (57.0)	
Adenocarcinoma	15 (8.9)	4 (5.0)	
Neuroendocrine tumors	6 (3.6)	4 (5.0)	
Mixed-cell type	13 (7.7)	6 (7.0)	
Missing	21 (12.4)	5 (6.0)	
Regimen of chemotherapy			
Gemcitabine and cisplatin	8 (38.1)	31 (44.0)	0.776
MVAC	0 (0.0)	3 (4.0)	
Taxane-based	0 (0.0)	1 (1.0)	
Carboplatin-based	7 (33.3)	19 (27.0)	
Others	6 (28.6)	16 (22.0)	
Bladder UC after RNU			
No	136 (81.9)	64 (84.0)	0.663
Yes	30 (18.1)	12 (15.0)	
Lymphadenectomy			
No	116 (68.6)	36 (47.7)	0.002^**^
Yes	53 (31.4)	40 (52.6)	
Follow-up (months)^b^ (median (IQR))	22.2 (6.2–54.1)	29.1 (11.1–5.9)	0.313

a Chi-squared test calculated for the different variables.

b Wilcoxon rank-sum test calculated for the difference in medians.

^*^p < 0.05; ^**^p < 0.01.

RNU, radical nephroureterectomy; CIS, carcinoma in situ; MVAC, methotrexate, vinblastine, doxorubicin (Adriamycin), cisplatin.

**Table 2:** Baseline demographic characteristics of patients with variant upper tract urothelial cancer (UTUC) undergoing radical nephroureterectomy (RNU) after matching.

Variables	UC with variants/No C/T (*N* = 50)	UC with variants/C/T (*N* = 50)	*p*-value^a^
	*N* (%)	*N* (%)	
Gender			
Men	23 (46.0)	23 (46.0)	1.000
Women	27 (54.0)	27 (54.0)	
Age			
<70	27 (54.0)	31 (62.0)	0.418
≥70	23 (46.0)	19 (38.0)	
Comorbidity			
Coronary artery disease	4 (8.0)	4 (8.0)	1.000
Arrhythmia	4 (8.0)	2 (4.0)	0.400
Hypertension	29 (58.0)	19 (38.0)	0.045^*^
Diabetes	15 (30.0)	9 (18.0)	0.160
Gouty arthritis	1 (2.0)	3 (6.0)	0.307
Gastrointestinal disorder	4 (8.0)	6 (12.0)	0.505
2nd malignancy (not urothelial cancer)	9 (18.0)	8 (16.0)	0.790
Tumor location			
Renal pelvis	26 (52.0)	19 (38.0)	0.369
Ureter	12 (24.0)	16 (32.0)	
Renal pelvis + ureter	12 (24.0)	15 (30.0)	
Tumor size			
<3 cm	12 (24.0)	20 (40.0)	0.086
≥3cm	38 (76.0)	30 (60.0)	
RNU histology			
Low grade	1 (2.0)	0 (0.0)	0.169
High grade	46 (92.0)	48 (98.0)	
Gx	3 (6.0)	0 (0.0)	
G2	0 (0.0)	1 (2.0)	
Multiplicity			
Not available	0 (0.0)	1 (2.0)	0.158
No	33 (67.3)	25 (50.0)	
Yes	16 (32.7)	24 (48.0)	
CIS			
No	36 (72.0)	38 (76.0)	0.648
Yes	14 (28.0)	12 (24.0)	
Lymphovascular invasion			
No	30 (60.0)	28 (56.0)	0.685
Yes	20 (40.0)	22 (44.0)	
Surgical margin			
Free	47 (94.0)	48 (96.0)	0.646
Positive	3 (6.0)	2 (4.0)	
Tumor necrosis			
No	29 (59.2)	28 (56.0)	0.749
Yes	20 (40.8)	22 (44.0)	
Synchronous bladder tumor			
No	40 (81.6)	41 (82.0)	0.999
Yes	9 (18.4)	9 (18.0)	
Pathological stage T			
pTis/pTa/pT0/pT1/pT2	7 (14.0)	7 (14.0)	1.000
pT3/pT4	43 (86.0)	43 (86.0)	
Pathological stage N			
pN0	15 (30.0)	13 (26.0)	0.842
pN+	9 (18.0)	11 (22.0)	
pNx	26 (52.0)	26 (52.0)	
eGFR			
≧60	15 (31.9)	21 (44.7)	0.203
<60	32 (68.1)	26 (55.3)	
Post-OP eGFR			
≧60	4 (9.1)	7 (22.6)	0.104
<60	40 (90.9)	24 (77.4)	
Histologic			
Sarcomatoid differentiation	8 (16.0)	10 (20.0)	0.692
Squamous cell carcinoma	19 (38.0)	25 (50.0)	
Adenocarcinoma	5 (10.0)	3 (6.0)	
Neuroendocrine tumors	3 (6.0)	3 (6.0)	
Mixed-cell type	7 (14.0)	4 (8.0)	
Missing	8 (16.0)	5 (10.0)	
Regimen of chemotherapy			
Gemcitabine and cisplatin	2 (20.0)	18 (40.0)	0.623
MVAC	0 (0.0)	2 (4.0)	
Taxane-based	0 (0.0)	1 (2.0)	
Carboplatin-based	4 (40.0)	13 (28.0)	
Others	4 (40.0)	11 (24.0)	
Bladder UC after RNU			
No	42 (84.0)	41 (82.0)	0.790
Yes	8 (16.0)	9 (18.0)	
Lymphadenectomy			
No	27 (54.0)	26 (52.0)	0.841
Yes	23 (46.0)	24 (48.0)	

a Chi-squared test calculated for the different variables.

b Wilcoxon rank-sum test calculated for the difference in medians.

^*^p < 0.05.

RNU, radical nephroureterectomy; CIS, carcinoma in situ; MVAC, methotrexate, vinblastine, doxorubicin (Adriamycin), cisplatin.

### Survival Analyses: Variant UTUC Without Chemotherapy vs. Variant UTUC With Chemotherapy

The median follow-up period was comparable between the vUTUC−C/T and vUTUC+C/T groups (22.2 and 29.1 months, respectively) (**Table 1**). In the univariate analyses, survival difference was only identified in overall survival (hazard ratio (HR), 0.527; 95% confidence interval (CI), 0.285–0.973; *p* = 0.041) (**Table 3**). However, in multivariate analyses, after adjusting with confounding factors selected with stepwise selection, significant survival benefit was found in disease-free survival (DFS) and cancer-specific survival (CSS) for the vUTUC+C/T patients (**Tables 4** and **5**). In brief, patients with variant histology who underwent adjuvant chemotherapy was associated with significant survival benefit in DFS (HR, 0.324; 95% CI, 0.155–0.677; *p* = 0.003) and CSS (HR, 0.454; 95% CI, 0.208–0.988; *p* = 0.047). LVI was the common independent risk factor for CSS and DFS. Positive surgical margin status is the independent risk factor for DFS (HR, 6.047; 95% CI, 1.554–23.53; *p* = 0.009).

**Table 3:** Univariate and multivariate regression overall survival (OS) analyses in patients with variant upper tract urothelial cancer (UTUC) undergoing radical nephroureterctomy (RNU).

Variables	Univariate analysis		Multivariate analysis	
	HR (95% CI)	*p*-value	HR (95% CI)	*p*-value
OS group				
UTUC with variants/no C/T	1	0.041^*^	1	0.070
UTUC with variants/C/T	0.527 (0.285, 0.973)		0.532 (0.301, 1.048)	
Hypertension	2.093 (1.128, 3.882)	0.019^*^	2.165 (1.152, 4.069)	0.016^*^

CI, confidence interval; HR, hazard ratio; OS, overall survival; C/T, chemotherapy.

^*^p < 0.05.

**Table 4:** Univariate and multivariate regression cancer-specific survival (CSS) analyses in patients with variant upper tract urothelial cancer (UTUC) undergoing radical nephroureterctomy (RNU).

Variables	Univariate analysis		Multivariate analysis	
	HR (95% CI)	*p*-value	HR (95% CI)	*p*-value
CSS group				
UTUC with variants/no C/T	1	0.090	1	0.047^*^
UTUC with variants/C/T	0.522 (0.247, 1.106)		0.454 (0.208, 0.988)	
Sex				
Men	1		1	
Women	0.546 (0.255, 1.166)	0.118	0.329 (0.329, 0.137)	0.013^*^
Lymphovascular invasion	2.482 (1.171, 5.259)	0.018^*^	3.761 (1.667, 8.485)	0.001^**^
Surgical margin	3.542 (1.061, 11.825)	0.040^*^	6.047 (1.554, 23.53)	0.009^**^
Hypertension	2.306 (1.077, 4.939)	0.031^*^		

CI, confidence interval; HR, hazard ratio; CSS, cancer-specific survival; C/T, chemotherapy.

^*^p < 0.05; ^**^p < 0.01.

**Table 5:** Univariate and multivariate regression disease-free survival (DFS) analyses in patients with variant upper tract urothelial cancer (UTUC) undergoing radical nephroureterctomy (RNU).

Variables	Univariate analysis		Multivariate analysis	
	HR (95% CI)	*p*-value	HR (95% CI)	*p*-value
DFS group				
UTUC with variants/no C/T	1	0.072	1	0.003^**^
UTUC with variants/C/T	0.555 (0.292, 1.054)		0.324 (0.155, 0.677)	
Tumor size				
<3 cm	1	0.025^*^		
≥3 cm	2.565 (1.129, 5.828)			
Lymphovascular invasion	2.556 (1.339, 4.878)	0.004^**^	2.665 (1.188, 5.975)	0.017^*^
Pathological stage N				
pN0	1		1	
pN+	3.118 (1.291, 7.528)	0.011^*^	3.374 (1.284, 8.864)	0.014^*^
pNx	1.187 (0.512, 2.752)	0.689	1.598 (0.652, 3.918)	0.306
Hypertension	1.975 (1.036, 3.768)	0.039^*^		

CI, confidence interval; HR, hazard ratio; DFS, disease-free survival; C/T, chemotherapy.

^*^p < 0.05; ^**^p < 0.01.

**Figure 2:**
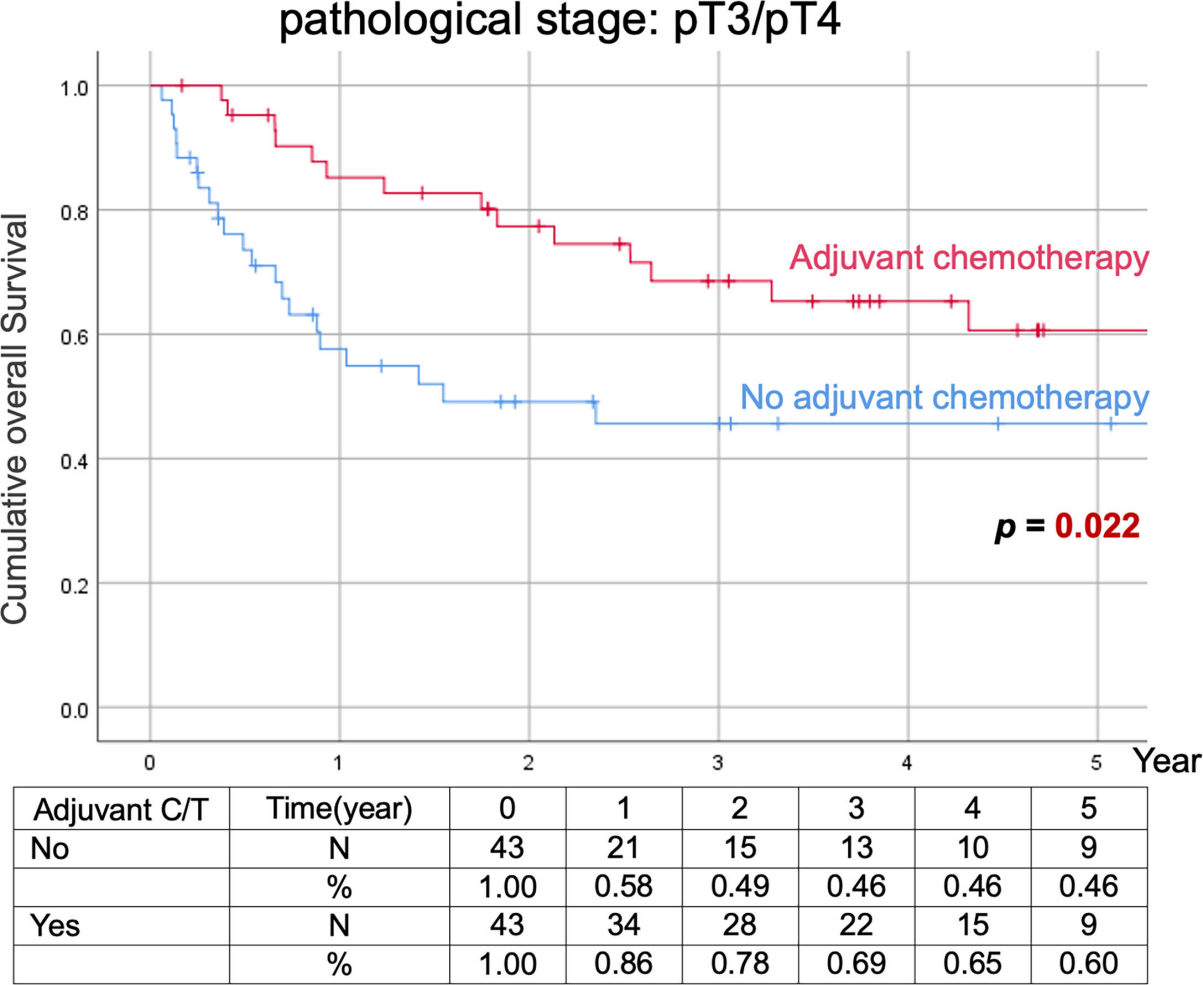
Kaplan–Meier analyses of overall survival in patients with advanced vUTUC with or without adjuvant chemotherapy.

**Figure 3:**
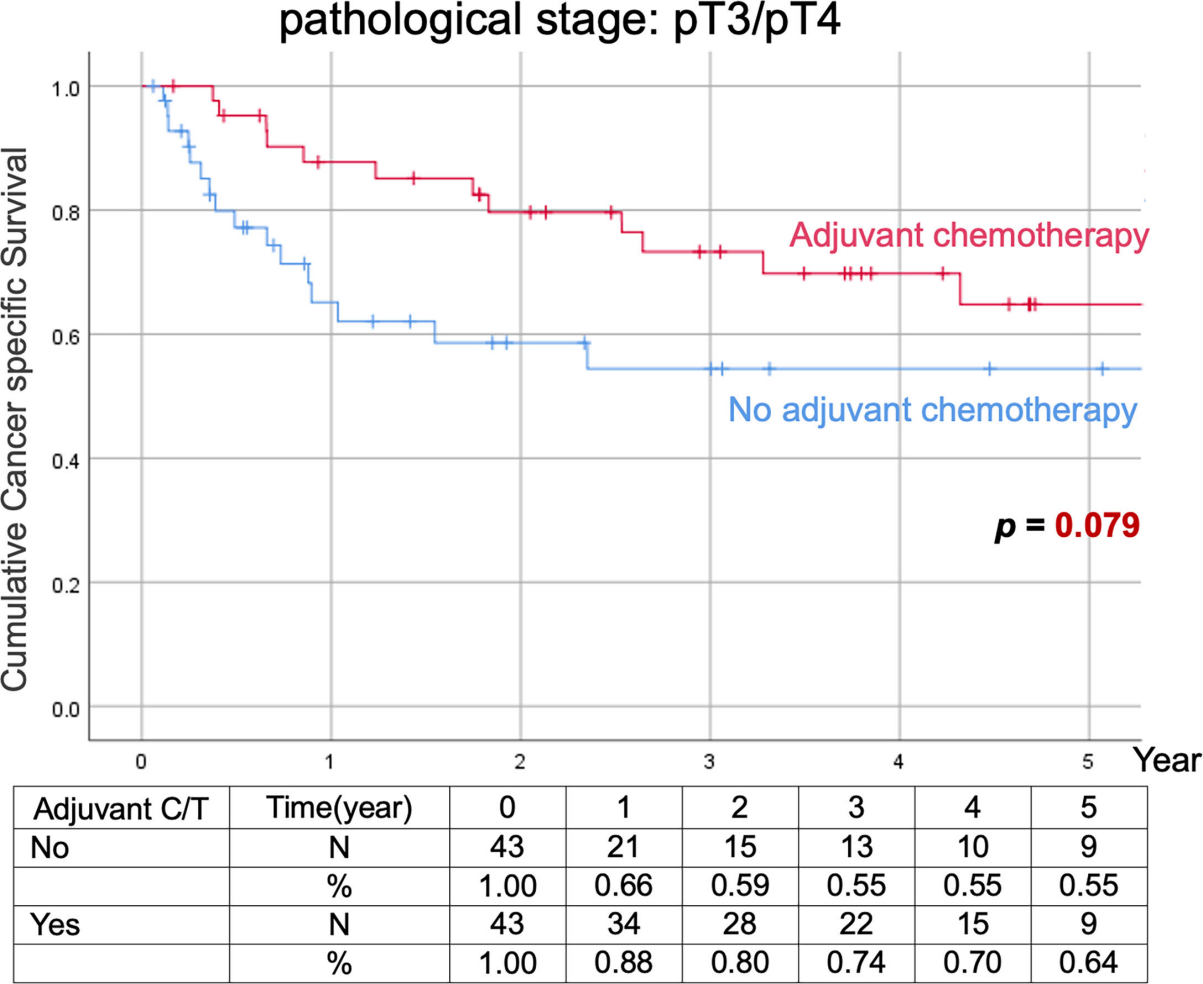
Kaplan–Meier analyses of cancer-specific survival in patients with advanced vUTUC with or without adjuvant chemotherapy.

**Figure 4:**
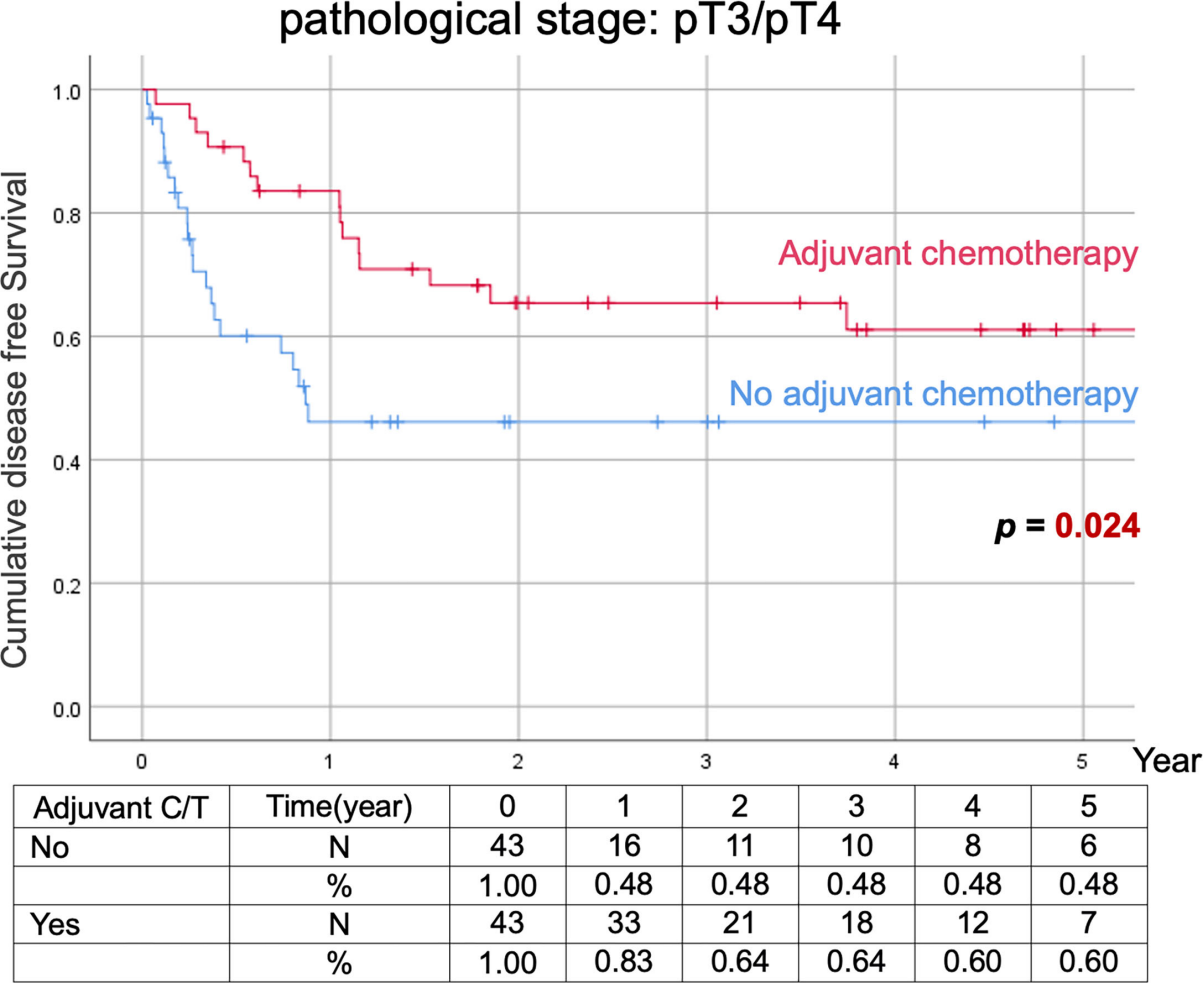
Kaplan–Meier analyses of disease-free survival in patients with advanced vUTUC with or without adjuvant chemotherapy.

Comparison of Kaplan–Meier estimated survival curves between vUTUC with and without adjuvant chemotherapy in localized vUTUC (pT0–T2) and advanced vUTUC (pT3–T4) disease after PSM. The survival benefit was observed in OS and DFS for advanced vUTUC disease with adjuvant chemotherapy (**Figures 2**–**4**).

## Discussion

UTUC is an uncommon malignancy in Western countries, and UTUC associated with variant histology is even rarer; with an overall incidence of 13.4% among the worldwide UTUC cohort (1). The variant histology UTUC is an important adverse prognostic factor affecting most survival domains of UTUC (1, 17, 18). The squamous or/and glandular variant histology was associated with an even worse CSS compared with the other UTUC variants (1). Therefore, vUTUC is a warning and prognostic indicator that could require upfront systemic therapy which usually means chemotherapy before or after RNU for UTUC. Based on recent evidence, perioperative chemotherapy (neoadjuvant or adjuvant) is beneficial in prolonging survivals of UTUC (19–21). Although perioperative chemotherapy had been proved beneficial in bladder UC variants, currently available literature failed to reveal its benefit in vUTUC (3, 4, 8, 22).

Current evidence regarding the efficacy of systemic chemotherapy on vUTUC remained scarce in the literature. As UTUC and bladder UC shared several histological and prognostic features, the regimen of chemotherapy for UTUC commonly follows the classic regimen for bladder urothelial carcinoma. Hence, most urologists/oncologists adapt classic chemotherapy regimens of bladder UC for vUTUC. Hajiran et al. conducted a cohort study to compare the result of cystectomy followed by neoadjuvant chemotherapy. The patients with variant histology (micropapillary, plasmacytoid, nested variant, sarcomatoid, or neuroendocrine variant histology), had a comparable response rate as the pure urothelial carcinoma cohort (23). A National Cancer Database study showed neoadjuvant chemotherapy was associated with significant pathological downstaging for all histological variants (sarcomatoid, micropapillary, squamous, neuroendocrine, and adenocarcinoma) and overall survival improvement for patients with variant bladder UC (24). In addition, MIBC with micropapillary, plasmacytoid variant, or squamous/glandular differentiation should be treated with neoadjuvant chemotherapy according to the recommendation of the 2020 EAU-ESMO Consensus Statements (25). Therefore, there is a clear role of chemotherapy in bladder UC with histological variants. Hence, the current cisplatin-based chemotherapy regimen is potentially effective in urothelial carcinoma with variant histology, as UTUC deserved a prospective randomized trial to confirm our speculation.

There was a national cancer database study that analyzed survivals of the UTUC with variant histology in the renal pelvis after surgery (26). Being restricted to the limited clinicopathological information in the cancer database, it only revealed the survival benefit of adjuvant chemotherapy in patients with pure UTUC but not for the patients with UTUC variants. Indeed, several limitations would hamper the power of cancer database study, such as significant variations in regimen and cycles of chemotherapy, comorbidity of patients, and intergroup bias of adverse histologic factors. Based on our findings, several important clinicopathological factors were independently associated with the survivals of the variant UTUC cohort (**Tables 3**–**5**). The important clinicopathological data that include comorbidities, surgical margin status, tumor size, tumor location, and lymphovascular invasion were generally not available in the cancer database study, therefore, limited the power of a national cancer database study. Hence, a comprehensive clinicopathological database could help in clarifying the real-world strategies of cancer treatment.

Murakami and their colleagues reported a multi-institutional study where those patients with UTUC with variant histology is an independent risk factor for recurrence-free survival but not for CSS (6). In addition, those vUTUC with pT3 or higher T stage and/or positive lymph node status were indicated for adjuvant chemotherapy. However, due to its retrospective study design and very-limited case number (37 UTUC cases with variant histology), significant selection bias and low power to reveal the real impact of adjuvant chemotherapy. To the best of our knowledge, our study is currently the limited PSM cohort study for the impact of chemotherapy on vUTUC. The current study did not identify the beneficial effect of adjuvant chemotherapy in CSS and DFS for vUTUC (**Tables 4** and **5**). In addition, the presence of LVI, positive lymph node status, or a positive surgical margin are independent risk factors for poor survival outcomes, and adjuvant chemotherapy should be considered in this vUTUC after RNU.

The impact of the subtypes of variant histology has been reported in the literature without a definite conclusion. The micropapillary, squamous, and/or glandular subtypes were potentially associated with a worse CSS among patients with UTUC in a prior meta-analysis (1). However, the variant histology of adenocarcinoma had been reported to be associated with a better OS compared with pure UTUC in a cancer database study (26). This inconclusive result could be derived from excluding metastatic disease during case enrollment, therefore, excluding advanced variant adenocarcinoma subtype, which is commonly an important factor of lethal disease (27). According to our analysis, different variant subtypes had comparable survival outcomes (**Supplementary Figure S1**). However, limited by the small case number in each subtype, the real impact of vUTUC subtypes should be clarified with a large-scale prospective study.

Lymphadenectomy has been recommended in RNU for UTUC for its potential survival benefit. In our matched cohort, 23 (46%) of 50 patients without adjuvant chemotherapy and 24 (48%) of 50 patients with chemotherapy underwent lymphadenectomy during RNU. As the role of extended lymphadenectomy remained controversial in UTUC treatment, extended or regional lymphadenectomy was only performed in selected cases who were clinically suspected of having advanced or nodal diseases; otherwise, lymphadenectomy was not performed in low-risk diseases. According to a large population cohort of 16,619 UTUCs, only 15.4% of cases underwent LND; therefore, the proportion of lymphadenectomy in our cohort is clearly not low when compared with the historical series (28).

Our cohort was extracted from the Taiwan UTUC registry which is a multicenter UTUC cohort that enrolled more than 4,000 UTUC cases in Taiwan. In the currently enrolled cohort, we identified 245 (7.2%) vUTUC among 3,043 Taiwan UTUC patients from 16 centers. The incidence of variant UTUC varied among centers in Taiwan; it generally ranges from 2% to 13% with only two exceptions (only two centers had a high incidence of 20% which accounted for only 15% of the vUTUC cohort). According to a recent meta-analysis, vUTUC generally accounted for about 13% of all UTUC worldwide, and the incidence varied significantly among different centers (1). Therefore, significant interobserver variation in making the diagnosis of vUTUC is common and clearly inevitable in a multicenter study. Histology review has been recommended for the multicenter study of vUTUC; however, relevant studies remained extremely scarce in the literature. Last year, we randomly enrolled 154 UTUC cases from the Taiwan UTUC registry for histology review (29). Based on our review, 7.8% and 30.5% variant UTUC were identified by the local or the reviewing pathologist, respectively. Only a slight interobserver agreement was achieved with a kappa value of 0.168. However, whether the vUTUC has an impact on disease outcome, according to the univariate analysis, the presentation of variant histology was the only risk factor of DFS in the local pathology reviewed cohort, but not in the review pathology cohort. This could relate to an overdiagnosis of clinically nonsignificant vUTUC in the review pathologist’s cohort, therefore, certainly underestimating the impact of vUTUC on disease outcome. In summary, whether histology review of vUTUC is beneficial in disease outcome prediction for multicenter study remained controversial and need a prospective large cohort study in the future.

## Limitations

This study has some limitations. First, the current study is still limited by the small sample size, the lack of the desired power level, and the effect size that does not correspond to the required sample size. Second, the pathology was not centrally reviewed; therefore, interobserver reporting bias was also considered one of the limitations. To minimize the impact of in-concordance of pathology between observers, we used a standardized histological report format which was approved by the Pathology Society of Taiwan based on the AJCC TNM staging system, and the principles of pathology management for urothelial cancer in the NCCN guidelines to ensure a standardized management protocol. In addition, genitourinary pathologists in Taiwan followed the same training program, specimen manipulation protocol, diagnostic criteria, standardized report template, and peer review system in each local institution to minimize the interobserver bias. Third, the retrospective nature of the study design is subject to selection bias. We attempted to account for this by the multi-institution enrollment, PSM, and multivariate Cox regression analyses with adjustment of confounding factors. In addition, cases with incomplete or missing information were excluded, except for some minor variables. Fourth, a lymphadenectomy was mainly performed in patients with clinical suspicious nodal diseases or advanced clinical stages, therefore, not routinely performed during RNU in Taiwan. Hence, the rate of pNx was as high as 45%~76%, which significantly undermined our ability to have further interpretation regarding the influence of the nodal status. Fifth, overall mortality and cancer-specific mortality could be partially overlapped in the primary comparison of matched cohorts. To minimize this effect, multivariate analysis was performed to adjust the confounders that could impact the survival. Finally, the lack of standard templates in reporting specific variant histology inevitably introduced further bias in the reported results (24).

## Conclusion

In summary, adjuvant chemotherapy following RNU significantly improved CSS and DFS in patients with UTUC with a variant histology in the current propensity score-matched study. Hence, the effect of adjuvant systemic chemotherapy deserves a further prospective, multi-institutional study to elucidate the optimal care for these rare and challenging patients.

## Data Availability Statement

The original contributions presented in the study are included in the article/**Supplementary Material**. Further inquiries can be directed to the corresponding author.

## Author Contributions

Conceptualization: H-YW and C-WL. Data curation: W-ML, H-LK, Y-HC, H-CW, I-HC, J-TL, C-YH, C-HC, J-ST, W-RL, Y-HJ, Y-KL, C-YT, S-DC, TH, AC, Y-CJ, I-SC, Y-TC, J-SC, B-JC, C-CY, WL, C-CW, C-SC, and H-YW. Formal analysis: Y-CT. Methodology: W-ML and H-LK. Supervision: J-TL, AC, and Y-CJ. Writing—original draft: Y-CT. Writing—review and editing: Y-CT. All authors listed have made a substantial, direct, and intellectual contribution to the work and approved it for publication.

## Conflict of Interest

The authors declare that the research was conducted in the absence of any commercial or financial relationships that could be construed as a potential conflict of interest.

## Publisher’s Note

All claims expressed in this article are solely those of the authors and do not necessarily represent those of their affiliated organizations, or those of the publisher, the editors and the reviewers. Any product that may be evaluated in this article, or claim that may be made by its manufacturer, is not guaranteed or endorsed by the publisher.
