# Supplementary figures and images for: Impact of Adjuvant Chemotherapy on Variant Histology of Upper Tract Urothelial Carcinoma: A Propensity Score-Matched Cohort Analysis
Source: Front Oncol. 2022 Apr 22;12:843715. doi: 10.3389/fonc.2022.843715 (PMC9072967; doi:10.3389/fonc.2022.843715)

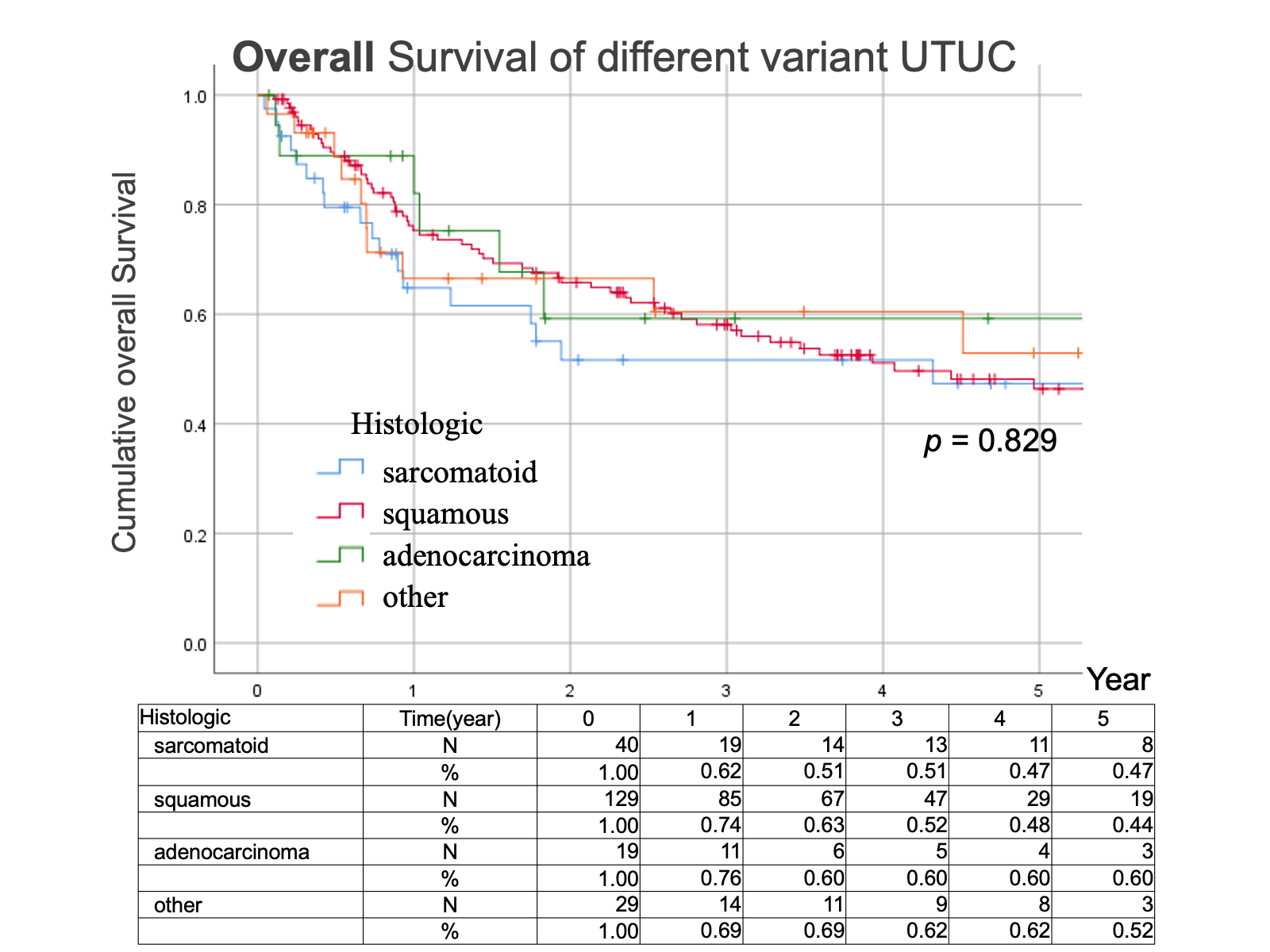

Supplement: Supplementary file 1 [file Image_1.tif]
